# Spanish Lymphoma Group (GELTAMO) guidelines for the diagnosis, staging, treatment, and follow-up of diffuse large B-cell lymphoma

**DOI:** 10.18632/oncotarget.25892

**Published:** 2018-08-17

**Authors:** Eva González-Barca, Mónica Coronado, Alejandro Martín, Carlos Montalbán, Santiago Montes-Moreno, Carlos Panizo, Guillermo Rodríguez, Juan Manuel Sancho, Andrés López-Hernández, on behalf of the Spanish Lymphoma Group (GELTAMO)

**Affiliations:** ^1^ Department of Hematology, Institut Català d' Oncologia and IDIBELL, L' Hospitalet de Llobregat, Barcelona, Spain; ^2^ Department of Nuclear Medicine, Hospital Universitario La Paz, Madrid, Spain; ^3^ Department of Hematology, Hospital Universitario de Salamanca, IBSAL, CIBERONC, Salamanca, Spain; ^4^ Department of Hematology, MD Anderson Cancer Center, Madrid, Spain; ^5^ Department of Pathology and Translational Hematopathology Lab, Hospital Universitario Marqués de Valdecilla/IDIVAL, Santander, Spain; ^6^ Department of Hematology, Clínica Universidad de Navarra and Instituto de Investigación Sanitaria de Navarra (IdiSNA), Pamplona, Spain; ^7^ Department of Hematology, Hospital Universitario Virgen de la Macarena, Sevilla, Spain; ^8^ Department of Hematology, ICO-IJC-Hospital Germans Trias i Pujol, Badalona, Barcelona, Spain; ^9^ Department of Hematology, Hospital Universitario Vall d'Hebron, Barcelona, Spain

**Keywords:** guidelines, DLBCL, risk assessment, treatment, DLBCL entities

## Abstract

Diffuse large B-cell lymphoma (DLBCL) accounts for approximately 30% of non-Hodgkin lymphoma (NHL) cases in adult series. DLBCL is characterized by marked clinical and biological heterogeneity, encompassing up to 16 distinct clinicopathological entities. While current treatments are effective in 60% to 70% of patients, those who are resistant to treatment continue to die from this disease.

An expert panel performed a systematic review of all data on the diagnosis, prognosis, and treatment of DLBCL published in PubMed, EMBASE and MEDLINE up to December 2017. Recommendations were classified in accordance with the Grading of Recommendations Assessment Development and Evaluation (GRADE) framework, and the proposed recommendations incorporated into practical algorithms. Initial discussions between experts began in March 2016, and a final consensus was reached in November 2017. The final document was reviewed by all authors in February 2018 and by the Scientific Committee of the Spanish Lymphoma Group GELTAMO.

## DIAGNOSIS

Surgical excision/incision biopsy of a node or affected extra-nodal tissue is the method of choice for diagnosis. Core needle biopsies may be performed in patients in whom surgical biopsy is excessively risky. Diagnosis is not based exclusively on the analysis of fine-needle aspirate, although this technique, together with adequate phenotyping, can be sufficient to confirm relapse [1].

Diagnosis of DLBCL is based on morphological, immunohistochemical, cytogenetic, and molecular analyses. DLBCL is characterized by the expression of pan B markers such as CD20, paired box protein 5 (PAX5), octamer transcription factor 2 (OCT2), and CD79a, which are detectable by immunohistochemistry (IHC) or flow cytometry [1]. Some entities, such as plasmablastic lymphoma, primary effusion lymphoma, anaplastic lymphoma kinase (ALK)-positive large B-cell lymphoma, and human herpes virus-8 (HHV-8)-positive DLBCL- not otherwise specified (NOS) show reduced expression of markers of B-cell differentiation and upregulation of plasma-cell differentiation antigens. In these cases, analyses of CD138, CD38, Ki67, MYC, multiple myeloma-1 (MUM1), ALK, HHV-8, and Epstein-Barr virus (EBV)-encoded small RNAs (EBERs) are recommended [1].

At least 2 molecular subtypes of DLBCL-NOS have been identified by gene expression profiling: the germinal center B-cell-like (GCB) and the activated B-cell-like (ABC), which differ in their prognosis [2]. These subtypes can be classified using a variety of immunohistochemical algorithms, which range in accuracy from 85% to 93% with respect to the gold standard (gene expression profiling) [3, 4]. The Hans algorithm demonstrated prognostic value for progression-free survival (PFS), although making therapeutic decisions based on these result is not currently supported by available evidence. Double protein (DP) expression of MYC and BCL2 is found in 20% to 35% of cases [5–7]. These cases are associated with worse survival in retrospective series, regardless of subtype (GCB *vs.* ABC). While it is recommended to note double MYC/BCL2 expression, there is insufficient evidence to suggest that an alternative therapeutic approach should be taken based on this parameter. CD30 expression is observed in 14% to 25% of cases, and is more common in certain entities, such as primary mediastinal large B cell lymphoma. It is recommended to report its expression at diagnosis [8, 9].

Studies have described cases characterized by concurrent rearrangements of *MYC* and *BCL2* and/or *BCL6* (double/triple hit: DH/TH) [5–7, 10]. These cases account for 3% to 14% of DLBCL and over 30% of cases formerly classified as B-cell lymphoma with features intermediate between DLBCL and Burkitt lymphoma. These cases are usually diagnosed in advanced stages with frequent involvement of the bone marrow and central nervous system (CNS), show a GCB phenotype and MYC protein overexpression in most instances, and have an adverse prognosis [11, 12]. Demonstration of *MYC, BCL2* and *BCL6* rearrangements can be done using FISH (fluorescent *in situ* hybridization) or conventional cytogenetic techniques. Cases with DLBCL morphology and DH/TH are now classified as High Grade B cell Lymphoma DH/TH, based on 2016 World Health Organization (WHO) classification update.

### Summary and recommendations for DLBCL diagnosis (Figure 1)

1. Excisional/incisional biopsy of the adenopathy or affected extranodal tissue is the method of choice for the diagnosis of DLBCL (Grade 1A).
2. Diagnosis of DLBCL is based on the results of histopathological, immunohistochemical, and cytogenetic/molecular studies. The combination of this information with clinical data allows classification according to the WHO (Grade 1A).
3. In all cases of DLBCL-NOS it is recommended to classify according to GCB/non-GCB phenotype, using the Hans algorithm (Grade 2B).
4. It is advisable to evaluate expression of MYC and BCL2 in DLBCL samples (Grade 2A).
5. Rearrangements of *MYC, BCL2,* and/or *BCL6* can be demonstrated using conventional cytogenetic techniques (karyotype) and/or FISH; this is a requirement for the diagnosis of cases of high-grade B cell lymphoma with DH/TH (Grade 2A).
6. It is recommended to establish the presence or absence of CD30 expression by IHC in DLBCL samples at diagnosis (Grade 2B).

**Figure 1:**
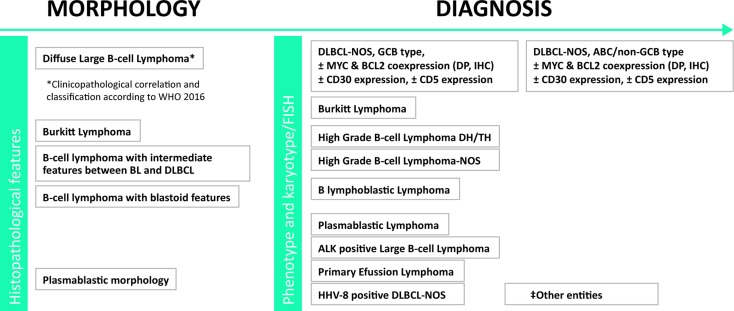
Histopathological diagnosis of DLBCL Tissue biopsy. (‡) Other entities: [T cell/histiocyte-rich large B-cell lymphoma, primary CNS large B-cell lymphoma, primary “leg type” large B-cell lymphoma, EBV-positive large B-cell lymphoma-NOS, DLBCL associated with chronic inflammation, lymphomatoid granulomatosis, primary mediastinal large B-cell lymphoma (thymic), Intravascular large B-cell lymphoma, Burkitt-like lymphoma with 11q aberration, B-cell lymphoma, Unclassifiable, with features intermediate between Hodgkin´s lymphoma and DLBCL (grey zone B-cell lymphoma), large B-cell lymphoma with IRF4 rearrangement]. DLBCL: large B-cell lymphoma; NOS: Not otherwise specified.

## STAGING AND RISK ASSESSMENT

A complete clinical history, including Eastern Cooperative Oncology Group (ECOG) functional status and the presence of B symptoms, and a complete physical examination, with particular emphasis on identifying lymphadenopathy and visceromegaly, are required, in addition to the following analyses: blood count; biochemical analysis including lactate dehydrogenase (LDH), beta2 microglobulin, urate, liver and renal function; proteinogram; and serology for hepatitis B (HBs antigen, anti-HBc), hepatitis C, and human immunodeficiency virus (HIV).

Fluorodeoxyglucose (FDG)-positron emission tomography (PET)/computed tomography (CT) is the test of choice for diagnosis of DLBCL extension, as it offers greater diagnostic accuracy than CT with contrast owing to its greater sensitivity for the detection of disease [13]. At present there is insufficient evidence to recommend the routine use of PET/CT with high-dose CT with intravenous contrast; the use of this procedure rarely results in changes to patient management and entails an increase in the radiation to which the patient is exposed. PET/CT is of limited value for the detection of CNS disease, and therefore magnetic resonance imaging (MRI) or cerebrospinal fluid (CSF) analysis is recommended in cases of suspected infiltration [13]. Staging by bone-marrow biopsy can be avoided in cases in which PET/CT reveals focal infiltration [13].

Measurement of ventricular ejection fraction is recommended in patients over 60 years of age and those with a history of cardiac disease.

The International Prognostic Index (IPI) is used in clinical practice to assess patient prognosis [14], with the limitation that it does not allow identification of very high-risk patients who have undergone immunochemotherapy [15]. Further independent studies are required to validate the proposed greater effectiveness of newer indices, including the National Comprehensive Cancer Network (NCCN)-IPI and GELTAMO-IPI [16, 17]. The inclusion of biomarkers and genes implicated in the biology of DLBCL has not significantly improved the prognostic accuracy of the IPI [18, 19]. The presence of concurrent *MYC* plus *BCL2* and/or *BCL6* rearrangements (DH/TH) confers an adverse prognosis, and is associated with an estimated 9-fold increase in the risk of mortality [11, 12, 20].

### Summary and recommendations for DLBCL staging and risk assessments

1. PET/CT is the standard method for the staging of patients with DLBCL, both in clinical practice and in clinical trials (Grade 1B).
2. Bone marrow biopsy can be avoided in cases in which PET/CT reveals bone infiltration (Grade 2C).
3. The IPI is the standard prognostic index used in clinical practice. It is useful as a prognostic tool to stratify patients in clinical trials (Grade 1A).
4. High grade B cell lymphoma with DH/TH (i.e. concurrent translocations of *MYC* and *BCL2*, and/or *BCL6*) has an adverse prognosis (Grade 2B).

## TREATMENT

### First-line therapy

First-line treatment can be stratified based on the extent of disease (limited stage or disseminated), the age of the patient, and their IPI score.

### Limited stage disease (Ann Arbor I-II)

In the pre-rituximab era, treatment with 3 cycles of CHOP (cyclophosphamide, doxorubicin, vincristine, and prednisone) followed by radiotherapy of the affected area was established as the standard treatment; this approach resulted in better PFS than 6 cycles of CHOP alone, although with longer follow-up no differences in OS were observed between patients who received radiotherapy and those who did not [21, 22]. In the rituximab era, 2 randomized trials have been performed. The Lymphoma Study Association (LYSA) group conducted a trial in which patients with non-bulky (<7 cm) limited-stage DLBCL were treated with 4 cycles of rituximab-CHOP (R-CHOP) and patients with high risk factors were treated with 2 additional cycles of R-CHOP. Those in complete metabolic remission (CMR) after 4 cycles of R-CHOP were randomized to 2 groups: involved-field radiation therapy (IFRT) or observation. No differences in event-free survival (EFS) (89% *vs.* 92%) or overall survival (OS) (92% *vs.* 96%) were observed [23]. Those who did not achieve CMR after 4 cycles of R-CHOP underwent 2 additional cycles of R-CHOP and IFRT. In the German UNFOLDER study, young patients with bulky limited-stage DLBCL were randomized to receive 6 cycles of R-CHOP followed by radiotherapy or not. The R-CHOP without radiotherapy arm was prematurely closed due to a high rate of relapse [24], and R-CHOP×6 + IFRT is therefore recommended for this group of patients.

### Disseminated disease (Ann Arbor III-IV)

#### Patients aged 60–80 years

The standard treatment in these patients is 6 to 8 cycles of R-CHOP every 21 days, based on the results of the French group trial that compared this treatment with CHOP without R and observed a significant increase in survival in the former group [25, 26]. Increasing dose density, which appeared to be beneficial in the RICOVER trial [14, 27], produced no benefits in 2 large randomized trials [28, 29]. Neither the addition of bortezomib to the R-CHOP-like regimen nor the substitution of rituximab with obinutuzumab (a type II anti-CD20 antibody) improved patient survival [30, 31]. Use of radiotherapy to treat extranodal lesions or bulky disease is controversial. However, its use is considered based on the findings of a cohort study in which patients that received radiotherapy had better progression-free survival (PFS) [32].

### Low or low-intermediate risk patients (IPI score, 0–2) aged <60 years

The standard treatment in these patients is 6 to 8 cycles of R-CHOP every 21 days, based on the results of the MiNT trial, which compared R-CHOP-like with CHOP-like regimens and found clear improvements in PFS and OS in patients in whom R was added [33, 34]. Patients in this trial with bulky disease received radiotherapy. A French trial of young patients with DLBCL and an age-adjusted (aa)-IPI = 1 compared the most intensive R-ACVBP (rituximab-doxorubicin, cyclophosphamide, vindesine, bleomycin and prednisone)/methotrexate (MTX)/R-ICE (rituximab-ifosfamide, carboplatin, and etoposide) followed by autologous-hematopoietic stem cell transplantation (auto-HSCT) regimen with R-CHOP, without using radiotherapy in any arm, and observed an increase in survival in patients treated with the intensive regimen, which was also associated with greater toxicity [35, 36].

### High-risk patients (IPI score, 3–5) aged <60 years

While there is no standard treatment for this group of patients, they are usually treated with 6 to 8 cycles of R-CHOP every 21 days. Dense-dose R-CHOP has shown no benefits [28]. In a phase II study, high-risk patients (IPI: 3–5; aaIPI: 2–3) were treated with DA-EPOCH-R [dose-adjusted-(etoposide, prednisone, vincristine, cyclophosphamide, doxorubicin-rituximab)] [37], and a randomized study (NCT00118209) comparing DA-EPOCH-R with R-CHOP is currently underway [38]. Some studies have investigated the use of high-dose chemotherapy followed by transplantation of autologous hematopoietic stem cells [39], but have reported contradictory results, and therefore this treatment cannot be recommended for all patients.

### Patients with comorbidities or aged >80 years

There is no standard treatment for patients of over 80 years of age. A geriatric assessment is recommended to identify “fit” patients. The combination of rituximab and polychemotherapy with doxorubicin induces complete remission and prolongs survival. Therefore, it is recommended to use conventional treatments such as R-CHOP, or an attenuated immunochemotherapy regimen (R-miniCHOP) [40]. In patients with cardiac pathologies adriamycin can be omitted (R-COP) or substituted with liposomal doxorubicin or other agents such as mitoxantrone, etoposide, or gemcitabine [41–43].

### Summary and recommendations for DLBCL first-line therapy (Figure 2)

1. R-CHOP × 4 cycles is recommended for patients with localized DLBCL without adverse prognostic factors (Grade 1A). For patients with high risk factors in CR by PET/CT, 2 additional courses of R-CHOP are recommended (Grade 1A). If PET/CT CR is not achieved, the recommendation is to give 2 additional cycles of R-CHOP and IFRT (Grade 1A).
2. R-CHOP × 6 cycles plus radiotherapy of the bulky area is recommended for patients with localized bulky DLBCL (>7 cm) (Grade 2B).
3. The standard treatment for patients with disseminated DLBCL aged 60 to 80 years is 6–8 cycles of R-CHOP administered every 21 days (Grade 1A). Radiotherapy for bulky disease or extranodal lesions may be of benefit in some patients (Grade 1B).
4. The standard treatment for patients with disseminated DLBCL aged less than 60 years with an IPI of 0–2 is 6 cycles of R-CHOP administered every 21 days (Grade 1A). Radiotherapy for bulky disease or extranodal lesions may be of benefit in some patients (Grade 1B).
5. There is no standard treatment for patients with disseminated DLBCL aged less than 60 years with a high IPI (IPI:3–5); these patients should be included in clinical trials. They are generally treated with 6 or 8 cycles of R-CHOP administered every 21 days (Grade 1B).
6. There is no standard treatment for patients with disseminated DLBCL aged over 80 years. It is recommended to use conventional treatments such as R-CHOP whenever possible, or to use attenuated doses of the same drugs (R-miniCHOP) (Grade 1C). Doxorubicin can be replaced with liposomal doxorubicin or other agents such as etoposide, mitoxantrone, or gemcitabine (Grade 1C).

**Figure 2:**
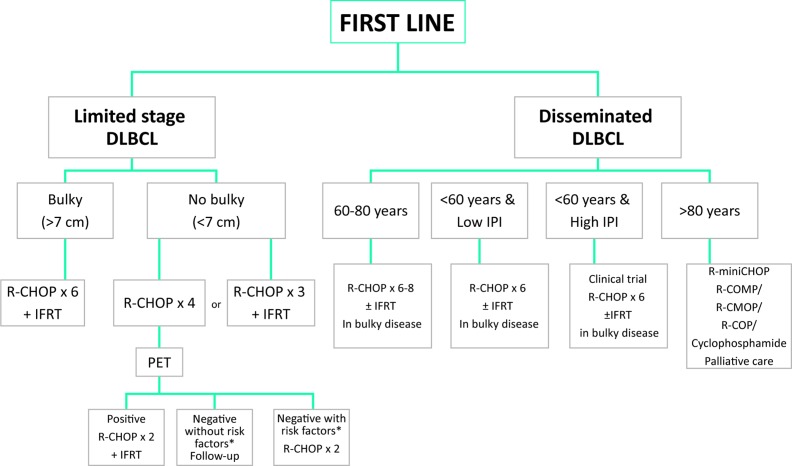
First line therapy ^*^Risk factors: High LDH level, Ann Arbor stage II, ECOG PS > 0, Age > 60 years. DLBCL: large B cell lymphoma; IFRT: involved-field radiation therapy; IPI: international prognostic index; R-CHOP: rituximab, cyclophosphamide, daunorubicin (doxorubicin), vincristine, prednisone; R-COMP: rituximab, cyclophosphamide, doxorubicin, vincristine and prednisone; RCOP: rituximab, cyclophosphamide, vincristine, prednisone.

### Salvage therapy

About 30-40% of patients have refractory disease or relapses after receiving first-line treatment. It is recommended whenever possible to repeat the biopsy of the tumor tissue to identify false-positive PET/CT or exclude other pathologies [13].

### High-dose chemotherapy

Salvage immunochemotherapy is the treatment of choice in young patients (<60–70 years) without comorbidities. In chemosensitive patients, the response should be consolidated with high-dose chemotherapy and auto-HSCT. The superiority of auto-HSCT over consolidation chemotherapy was established in the international randomized PARMA study in the 1990s [44]. No such studies have been performed in the rituximab era. No specific salvage regimen has demonstrated superiority over others. In the international CORAL study, R-DHAP (rituximab-dexamethasone, cytosine arabinoside, and cisplatin) and R-ICE (rituximab-ifosfamide, carboplatin, etoposide) showed similar efficacy, but the former was associated with greater renal toxicity and thrombopenia [45]. In the randomized NCIC-CTG LY.12 international trial, in which patients received R-GDP (rituximab-gemcitabine, cisplatin, and dexamethasone) or R-DHAP, both regimens showed similar efficacy, but R-DHAP resulted in greater toxicity (febrile neutropenia and thrombopenia) [46]. However results are very poor with any of the aforementioned regimens, with PFS around 20% [47]. Therefore, the best option for these patients is to enroll them in clinical trials of regimens that include new drugs. In this context, the substitution of rituximab with ofatumumab showed no benefit in a randomized study [48].

The main prognostic factor at the time of transplantation is lymphoma status, as determined by PET/CT, since patients with CMR have significantly better survival than patients in partial remission (PR) (PFS: 72%–87% *vs.* 18%–49%) [49, 50]. There are no randomized studies demonstrating the superiority of any conditioning regimen. The BEAM (BCNU, etoposide, cytarabine and melphalan) regimen is the most widely used in Europe. The incorporation of radioimmunotherapy into the conditioning regimen showed no superiority in the only phase III clinical trial published to date [51]. Maintenance rituximab after auto-HSCT also showed no benefits in the CORAL study [52]. Several retrospective studies have reported that allogeneic hematopoietic stem cell transplantation (allo-HSCT) may be effective in patients with relapse or progression after multiple lines of treatment, including auto-HSCT [53–55]. Given that myeloablative regimens are associated with a very high non-relapse-related mortality rate (40%–50%), it seems advisable to use non-myeloablative regimens (mortality: 20%–30%; PFS: 20%–40%). The status of the lymphoma is the most important prognostic factor, with very poor results in patients with chemorefractory disease [53–55]. In high-risk patients in PR after salvage therapy, allo-HSCT may be an alternative to auto-HSCT [56]. In controlled studies, allo-HSCT could be also an alternative to auto-HSCT [57, 58], for patients with very high-risk features, such as primary refractory disease.

### Non-auto-HSCT candidates

Evidence supporting the efficacy of salvage regimens in these patients is based on phase II studies. There is no standard regimen established and durable remissions are uncommon.

R-GemOx (rituximab-gemcitabine, oxaliplatin) results in overall response rates (ORR) of about 60% with a 1-year OS rate of 45% and a PFS of around 25% at 12 months [59, 60]. While results obtained with R-bendamustine (rituximab-bendamustine) in phase II trials vary significantly, this regimen has an acceptable toxicity profile and is relatively efficacious (ORR > 30%) in frail patients [61]. However, whenever possible these patients should be enrolled in clinical trials.

### Third-line and later lines of therapy

Third-line therapy is an option in patients who do not respond to salvage treatment. The scientific evidence supporting the efficacy of third-line therapy is lower than that published for previous lines of therapy. In some cases patients may be treated with second-line polychemotherapy regimens not used previously. A study comparing pixantrone monotherapy with other chemotherapeutic agents in monotherapy in relapsed patients that previously received a median of 3 lines of therapy reported overall response rates of 40% for pixantrone versus 14.3% for the comparator arm [62]. Lenalidomide in monotherapy or combined with rituximab shows efficacy in this situation, producing an overall response rate of around 30%, with a relatively short-duration response [63, 64]. Despite the higher ORR observed for the non-GCB subtype, no differences in PFS or OS were detected between the GCB and non-GCB subtypes [65]. There are no clinical trial data to indicate which other drugs in monotherapy (gemcitabine, oxaliplatin, etoposide, mitoxantrone, vinorelbine) are most appropriate in each case. These treatments have been used in comparator arms in several phase II clinical trials, and have shown poor efficacy in terms of ORR (usually <20%), but some clinical benefits [62]. Efficacy data are available for 2 treatment regimens at metronomic doses: celecoxib, methotrexate, and cyclophosphamide, which produced an ORR of 30% [66], and the PEP-C (prednisone, etoposide, procarbazine- cyclophosphamide) regimen, which produced a response in 3 out of 9 patients with DLBCL, with high toxicity [67]. Inclusion of these patients in clinical trials is recommended wherever possible.

### Summary and recommendations for DLBCL salvage therapy (Figure 3)

1. In refractory disease or relapse, it is advisable to repeat the tumor biopsy before proceeding to second-line treatment (Grade 1B).
2. The salvage regimen with the most favorable toxicity profile is R-GDP, although other regimens such as R-ESHAP, R-DHAP, and RICE have shown similar efficacy (Grade 1B). Patients who achieve at least PR following salvage therapy should undergo auto-HSCT with BEAM or similar conditioning regimen (Grade 1B).
3. Allogeneic transplantation is an option in patients that relapse after auto-HSCT (Grade 1C).
4. In patients with refractory or relapsing DLBCL who are not candidates for auto-HSCT, moderate-to-low intensity regimens using R-GemOx or R-bendamustine are recommended (Grade 1B).
5. There are no specific recommendations for third or later lines of therapy. Pixantrone has demonstrated superiority over other drugs in monotherapy in relapsed patients (Grade 1B).

**Figure 3:**
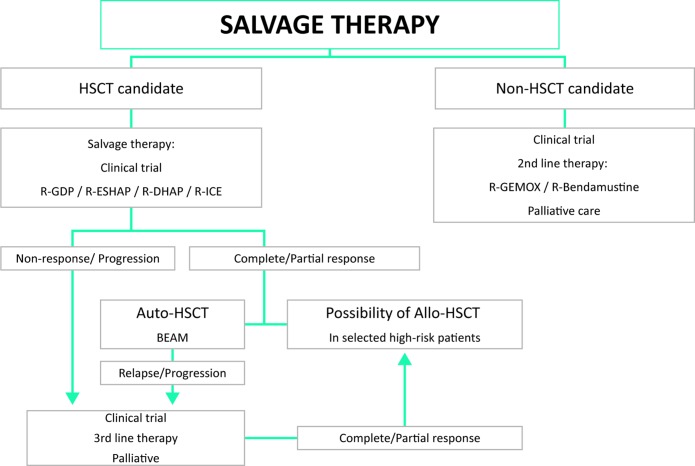
Salvage therapy BEAM: carmustine, etoposide, cytarabine, melphalan; HSCT: hematopoietic stem cell transplantation; R-DHAP: rituximab, dexamethasone, cytarabine, cisplatin; R-ESHAP: rituximab, etoposide, methylprednisolone; cytarabine; cisplatin; R-GDP: rituximab, gemcitabine, cisplatin, dexamethasone; R-ICE: rituximab, ifosfamide, carboplatin, etoposide.

## RESPONSE EVALUATION AND FOLLOW-UP

PET/CT at the end of treatment is the best instrument for assessing the patient's response. It is recommended to apply the criteria of the Lugano classification system, using the Deauville 5-point scale. The presence of a residual mass in the context of CMR is not a criterion for active disease. In cases of metabolically active residual disease, it is advisable to perform a biopsy if salvage therapy is considered [13, 68].

Interim PET (iPET)/CT is used to assess the early response, but there are some problems due to standardization regarding the technique itself and its interpretation [13]. CMR on iPET carries an excellent prognosis. Studies evaluating intensification treatment based on iPET have shown no benefit over R-CHOP [69, 70]. Therefore, iPET is not recommended as a basis for altering treatment outside of clinical trials.

After achieving CMR, clinical follow-up, with clinical history, physical examination, and laboratory work-up, should be performed every 3 to 4 months for the first 2 years and every 6 months for the following 3 years [13]. This should be followed by annual clinical follow-up to assess potential late toxicity and the possible development of second neoplasms. There is no evidence to suggest that routine CT or PET/CT provides any advantage over clinical follow-up assessment [13]. Most relapses occur outside of planned follow-up visits and only 1%–2% are detected in imaging tests [71], which provide no advantages for early detection of relapse and have no impact on survival.

### Summary and recommendations for DLBCL response evaluation and follow-up

1. PET/CT is the standard imaging modality used to assess the response at the end of treatment (Grade 1A).
2. Evaluation of the response should be carried out visually using the Deauville scale (Grade 2B).
3. In cases of metabolically active residual disease, it is advisable to perform a biopsy if salvage therapy is considered (Grade 2C).
4. In clinical practice, iPET is not recommended as a basis for changing treatment, unless there is clear evidence of progression, and should be reserved for clinical trials (Grade 2C).
5. The routine use of imaging tests in the follow-up of patients with DLBCL in complete remission is discouraged (Grade 1A).

## SPECIAL SITUATIONS

### CNS prophylaxis

This topic has been extensively discussed in recent specific guidelines published by the GELTAMO group [72].

### Tumor lysis syndrome prophylaxis

Risk factors for tumor lysis syndrome (TLS) include bulky tumor burden, a high proliferative index, pre-existing renal failure, advanced age, and treatment with highly active cell-cycle-specific agents. Prophylaxis with allopurinol is recommended in patients at intermediate risk of TLS. Treatment should be maintained for up to 7 days [73]. In high-risk patients (bulky disease [≥10 cm], LDH over twice the upper limit of normal, patients in the “*MYC*-driven” subgroup, and patients with renal impairment) [73, 74], TLS prophylaxis with hydration and rasburicase is recommended [75]. The volume infused is not specified, but 3 L/24 hours in adults seems a reasonable estimate. The standard dose of rasburicase is 0.2 mg/kg, for 5–7 days. However, studies have reported the use of lower doses, such as 0.1 mg/kg/day or 0.15 mg/kg/day, with a treatment duration adjusted to the patient's evolution, and the majority of patients only required a single dose [74, 76]. Concomitant use of rasburicase and allopurinol is not recommended, since the latter can reduce the efficacy of rasburicase.

### Summary and recommendations for DLBCL TLS prophylaxis

1. Patients with low risk TLS should be managed by monitoring water balance and laboratory parameters, and only the administration of allopurinol should be considered (Grade 2C). Intermediate-risk patients should be treated with allopurinol (7 days) and hydration (Grade 2C). High-risk patients should be treated with rasburicase and hyperhydration (Grade 1B).

## SPECIAL SUBTYPES

### Plasmablastic lymphoma

Plasmablastic lymphoma is mainly associated with human immunodeficiency virus (HIV) infection, but it has also been described in patients with other types of immune compromise and in immunocompetent patients [77, 78]. It usually presents with extranodal involvement, affecting the oral cavity in almost half of all cases. The prognosis is poor, with median survival ranging from 8–15 months [78]. There is no standard treatment. Some reviews recommend more intensive chemotherapy regimens such as EPOCH (etoposide, doxorubicin, and cyclophosphamide with vincristine and prednisone) or hyper CVAD/MA (hyperfractionated cyclophosphamide, vincristine, doxorubicin, and dexamethasone alternating with methotrexate and cytarabine), especially given the role of *MYC* alterations in the pathogenesis of this lymphoma [77]. However, 2 retrospective studies found no survival benefit of intensive regimens over CHOP in HIV patients [79, 80]. The use of CNS prophylaxis has not been systematically evaluated, but seems advisable given the frequent extranodal involvement, the high proliferative index, and the presence of *MYC* rearrangements in many cases [77]. Loco-regional radiotherapy has been used in some cases, although its role has not been fully established [77]. The use of auto-HSCT remains controversial: while some authors have proposed it as first-line consolidation therapy [81], others propose that it should only be used in cases that fulfill risk criteria [82, 83]. The incorporation of new drugs has produced promising results in recent studies. These include thalidomide, lenalidomide, and in particular the proteasome inhibitor bortezomib, which when combined with DA-EPOCH [84] or with CHOP [85] followed by auto-HSCT produced long-term remission.

### Primary mediastinal B-cell lymphoma (PMBCL)

This predominantly affects young adults (mainly women aged 30–40 years) and usually manifests as a bulky mediastinal mass, often with pleural or pericardial effusion and superior vena cava syndrome [86, 87]. Although there are few prospective studies, some sub-analyses such as the MinT trial [88] (with 11% of patients with PMBCL) concluded that the addition of rituximab to CHOP-like (CHOP or CHOEP) regimens significantly increased the CR rate and increased 3-year PFS (78% *vs.* 52%, *p* = 0.01) but not OS (88.5% *vs.* 78.2%, *p* = 0.15), possibly due to the small number of patients in this subgroup. Other retrospective studies have compared R-CHOP with CHOP regimens, and found that the former provides better outcomes [89]. A prospective phase II NCI study in which patients received infusional DA-EPOCH-R reported a CR rate of 94%, a 5-year EFS of 93%, and an OS of 97%. Only 2 patients required radiotherapy [86]. However, to date, no prospective study has directly compared DA-EPOCH-R with R-CHOP.

The role of radiotherapy is controversial. In a series that included patients treated with R-CHOP, CHOP, and intensive regimens such as MACOP-B (methotrexate, doxorubicin, cyclophosphamide, vincristine, prednisone, and bleomycin) and VACOP-B (etoposide, doxorubicin, cyclophosphamide, vincristine, prednisone, and bleomycin), the addition of radiotherapy had no effect on PFS or OS [90]. The use of DA-EPOCH-R provides good results without the need for radiotherapy [86]. The definitive role of radiotherapy may be clarified upon publication of the results of the International Extranodal Lymphoma Study Group (IELSG-37) study, in which patients who achieve CMR are randomized to radiotherapy or observation arms (https://www.clinicaltrials.gov/; NCT01599559). Frontline consolidation treatment with auto-HSCT does not provide any benefit [91]. Refractoriness and relapse are usually associated with an unfavorable prognosis [92]. Treatment is usually based on salvage immunochemotherapy and consolidation with auto-HSCT, and the results obtained tend to be poorer than those observed for DLBCL-NOS [93].

### High-grade lymphoma with *MYC* and *BCL2* and/or *BCL6* rearrangement (double/triple hit)

This type of lymphoma usually presents at advanced age, with high LDH and extranodal involvement, especially CNS infiltration [94]. It is important to differentiate DH/TH lymphomas from those with DP expression of MYC and BCL2. In patients with DH/TH lymphomas, standard treatment based on R-CHOP offers poor results with a CR rate of 40% and OS at 2 years of 35%–40% [5, 20, 95, 96]. Most intensive chemotherapy regimens, in particular DA-EPOCH-R, appear to achieve better CR and PFS than R-CHOP [5, 20, 97]. However, in a meta-analysis [98] of 11 studies and a total of 394 patients treated with R-CHOP (*n* = 180), R-EPOCH (*n* = 91), and R-hyper CVAD/R-MA or R-CODOX-M/R-IVAC (rituximab-cyclophosphamide, doxorubicin, vincristine, methotrexate/ifosfamide, etoposide, high-dose cytarabine) (*n* = 123), the risk of progression was significantly lower in R-EPOCH- versus R-CHOP-treated patients, but no significant differences in OS were observed between the 3 regimens, with median OS ranging from 20–30 months. Retrospective studies have corroborated the poor results obtained with auto-HSCT in patients with DH lymphoma [99]. Inclusion of these patients in clinical trials is recommended. CNS prophylaxis is highly recommended as it appears to be associated with improved survival [100].

### DLBCL in HIV patients

DLBCL is an AIDS-defining neoplasm. The prognosis of patients with DLBCL before the antiretroviral (ART) era was poor, but since the introduction of highly active ART (HAART), survival is similar to that of non-HIV-infected patients, using the same immunochemotherapy regimens [101, 102]. In these patients adequate antimicrobial prophylaxis and the use of G-CSF as primary prophylaxis for febrile neutropenia is recommended. Some studies suggested a higher incidence of CNS infiltration in the pre-ART era [103], but with HAART there has been a decrease in CNS infiltration, and therefore the recommendations for prophylaxis of CNS involvement are the same as for immunocompetent patients.

### Post-transplant lymphoproliferative disorder

Post-transplant lymphoproliferative disorder (PTLD) is one of the most serious complications that can affect patients undergoing solid-organ transplantation or allogeneic stem cell transplantation. Incidence ranges from 1%–20%. As a result of the immunodepressive state induced to prevent rejection, T lymphocytes fail to adequately control the proliferation of EBV-infected B lymphocytes, increasing the likelihood of errors during gene rearrangement, activation of oncogenes, inactivation of suppressor genes, and the development of lymphoma. The WHO classification of PTLD describes 3 types: early lesions which are usually polyclonal; polymorphic PTLD; and monomorphic PTLD, which is characterized by conventional-like lymphomas and is usually monoclonal. Most cases of monomorphic PTLD are large B-cell lymphomas. Around a half of all cases appear within 1 year of transplantation. Late-onset cases appear similar to conventional lymphoma, although they are more frequently characterized by extranodal, especially gastrointestinal, involvement. PTLD is staged by PET/CT [13, 68]. In cases of suspected PTLD, the first step is to reduce immunosuppression without endangering the viability of the graft [104]. This achieves a response rate of 20%–50%, particularly in cases of polymorphic PTLD. Localized PTLD can be eradicated by surgical resection or radiotherapy. In cases of disseminated PTLD with CD20 expression, rituximab monotherapy is the treatment of choice, with CR rates of 60%–70% [105, 106]. If CR is not achieved, a short course of 3–4 cycles of immunochemotherapy (*e.g.* R-CHOP) is recommended. Patients who fail to respond to other therapies have been treated with infusions of specific allogeneic cytotoxic T lymphocytes against EBV, resulting in a CR rate of 45% [107, 108]. This strategy cannot be applied in all centers due to the high associated costs and the specialized equipment required. In cases of transplant recipients at high risk of PTLD (*e.g.* recipients of haploidentical hematopoietic stem cell transplants, recipients of multivisceral and heart/lung transplants, and patients who are EBV-negative before transplantation) EBV copy number needs to be monitored frequently during the first 6 months to initiate pre-emptive therapy (1–4 doses of weekly rituximab, depending on the evolution of the viral load) while reducing immunosuppression [109]. There is no standard threshold for treatment initiation. Some authors recommend treatment if copy number exceeds 1000, while others propose thresholds of 10,000 or even 40,000.

### Summary and recommendations for special DLBCL subtypes (Figure 4)

1. There is no standard treatment for plasmablastic lymphoma. DA-EPOCH is recommended especially in cases with *MYC* rearrangement (Grade 2C).
2. In patients with primary mediastinal lymphoma, treatment is based on immunochemotherapy with rituximab (Grade 1A). More intensive regimens such as DA-EPOCH-R may be more effective, particularly in cases with unfavorable risk factors (Grade 2B). The effect of complementary radiotherapy in patients who have not achieved CMR is unclear. Whenever possible, it is recommended to repeat the biopsy given the high rate of false positives and the limited value of PET/CT in these cases (Grade 1B).
3. There is no standard therapy for DH/TH lymphomas; these patients should be included in clinical trials. Immunochemotherapy using intensive regimens (*e.g.* DA-EPOCH-R) or others, such as those used in Burkitt lymphoma, are recommended (Grade 1B). No data have demonstrated a benefit of auto-HSCT as first-line consolidation therapy in patients with CR (Grade 2C). CNS prophylaxis is recommended (Grade 1C).
4. In HIV patients, the treatment of DLBCL is based on immunochemotherapy, preferably with standard R-CHOP (Grade 2B). Multidisciplinary management is recommended to determine the optimal antiretroviral treatment regimen (Grade 2B). Primary prophylaxis with G-CSF is recommended (Grade 2B). In patients with chemosensitive relapse, the same salvage treatments as those used in immunocompetent patients are recommended, followed by consolidation with auto-HSCT (Grade 1B).
5. In PTLD patients, the first recommendation is to reduce immunosuppression (Grade 1B), followed, in CD20-positive cases, by weekly rituximab treatment (4–8 doses). If CR is not achieved patients may require 3–4 cycles of R-CHOP (Grade 1B). In transplant patients with a high risk of PTLD, monitor EBV copy number during the first 6 months, and institute pre-emptive therapy if copy number increases, reducing immunosuppression and administering 1–4 weekly doses of rituximab, depending on the evolution of the viral load (Grade 1C).

**Figure 4:**
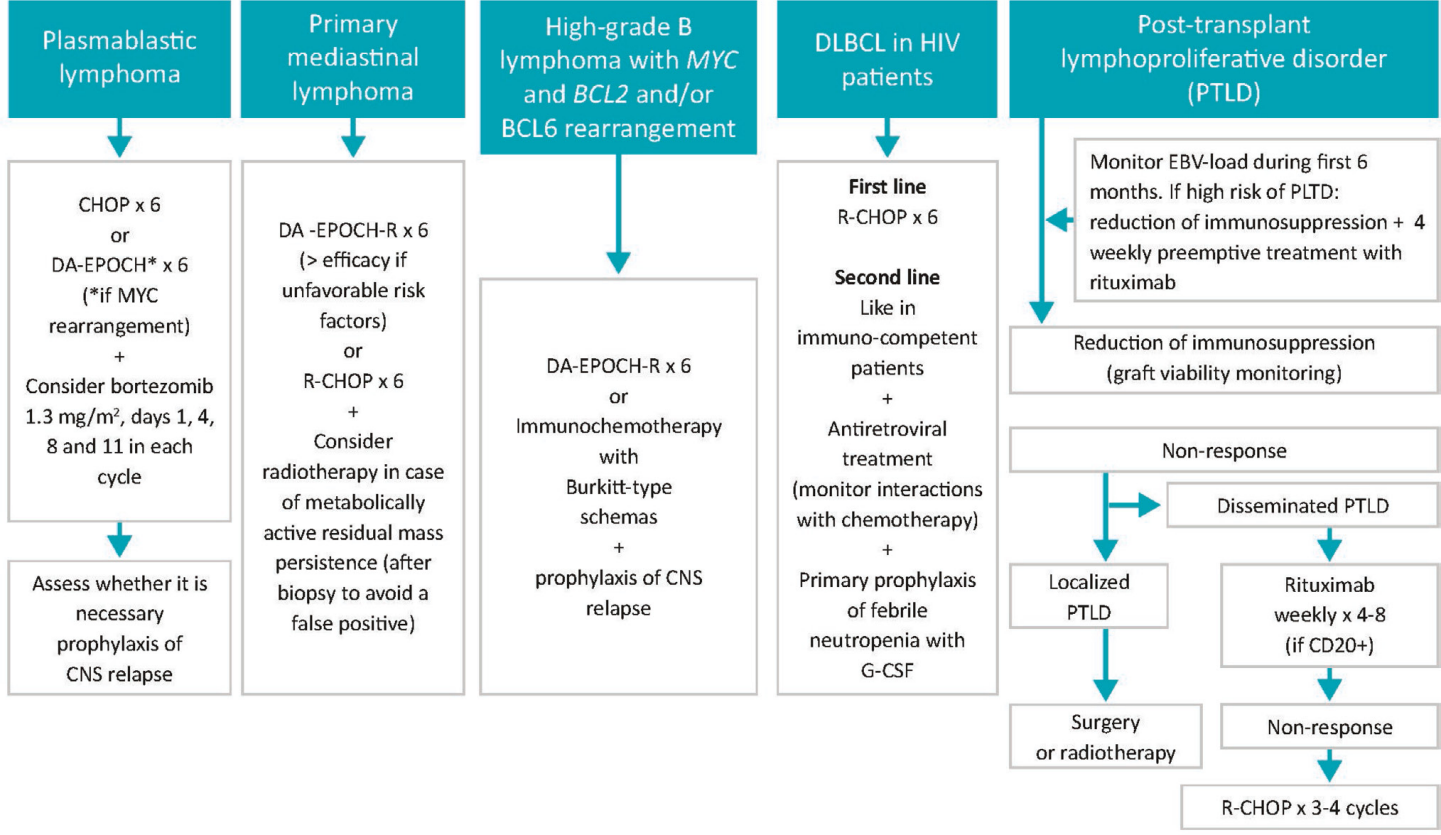
Treatment for special DLBCL subtypes CNS: central nervous system; DA-EPOCH-R: dose-adjusted, etoposide, prednisone, vincristine, cyclophosphamide, daunorubicin (doxorubicin), rituximab; EBV: Epstein-Barr virus; G-CSF: granulocyte colony stimulating factor; PTLD: post-transplant lymphoproliferative disorder; R-CHOP: rituximab, cyclophosphamide, daunorubicin (doxorubicin), vincristine, prednisone.
